# Beyond Domestic Cats: Environmental Detection of *Sporothrix brasiliensis* DNA in a Hyperendemic Area of Sporotrichosis in Rio de Janeiro State, Brazil

**DOI:** 10.3390/jof8060604

**Published:** 2022-06-04

**Authors:** Fernando Almeida-Silva, Vanessa Brito de Souza Rabello, Bruno de Souza Scramignon-Costa, Rosely Maria Zancopé-Oliveira, Priscila Marques de Macedo, Rodrigo Almeida-Paes

**Affiliations:** 1 Laboratório de Micologia, Instituto Nacional de Infectologia Evandro Chagas, Fundação Oswaldo Cruz, Rio de Janeiro 21040-900, Brazil; fernando.almeida@ini.fiocruz.br (F.A.-S.); vanessa.brito@ini.fiocruz.br (V.B.d.S.R.); bruno.costa@ini.fiocruz.br (B.d.S.S.-C.); rosely.zancope@ini.fiocruz.br (R.M.Z.-O.); 2 Laboratório de Pesquisa Clínica em Dermatologia Infeciosa, Instituto Nacional de Infectologia Evandro Chagas–Fundação Oswaldo Cruz, Rio de Janeiro 21040-900, Brazil; priscila.marques@ini.fiocruz.br

**Keywords:** soil, molecular biology, PCR, *Sporothrix*

## Abstract

In Brazil, sporotrichosis has transitioned from a rural to urban disease, driven by a shift in the initiation of infection from the accidental inoculation of organic matter to the traumatic implantation of the fungus by cats. Since the emergence of zoonotic sporotrichosis caused by *Sporothrix brasiliensis*, investigations have largely ignored the environmental habitat of the pathogen due to its association with domestic cats. Therefore, we investigated 18 environmental samples collected from rural areas of two cities where zoonotic sporotrichosis is endemic, but where domestic cats are scarce. We utilized traditional culture methods, and samples were also examined with two molecular methods used for the clinical diagnosis of sporotrichosis: a nested-PCR targeting the ITS region and a species-specific PCR targeting the calmodulin gene. No *Sporothrix* colonies were identified by traditional culture methods. However, the nested-PCR and the species-specific PCR for *S. brasiliensis* were positive for 18 and 5 samples, respectively. Sequencing revealed that positive results with the nested-PCR were due to non-specific amplification of other Ophiostomatales DNA, rather than *Sporothrix* spp. Three of the five amplicons from the species-specific PCR were suitable for sequencing and confirmed the presence of *S. brasiliensis* DNA. Hence, we confirmed that *S. brasiliensis*, as with other *Sporothrix* species, has an environmental habitat. Our findings underscore the challenges of nested-PCR for *Sporothrix* environmental studies and highlight that sequencing must follow PCR protocols to definitively identify *Sporothrix* spp. in environmental samples.

## 1. Introduction

Sporotrichosis is a subcutaneous mycosis with a worldwide distribution and broad spectrum of disease manifestations [1]. In immunocompetent people, sporotrichosis is typically a benign disease, and the most severe forms are usually observed in people living with HIV/AIDS (PLWHA) [2] and in individuals with other underlying immunosuppressive conditions [3,4].

Initially, sporotrichosis was known as rose gardener’s disease [1,5]. Its classical transmission is characterized by traumatic inoculation of contaminated material in the subcutaneous tissue, and the injury is frequently associated with vegetation, wood and soil manipulation [5,6]. In this scenario, the major at-risk individuals are those involved with horticulture, farming, mining and wood exploration [7]. This transmission form is becoming relatively less frequent in Brazil, where zoonotic transmission prevails [8,9,10,11]. 

Over the past 20 years, cats have played an important role in the transmission of sporotrichosis in Rio de Janeiro state, Brazil [1]. More recently, zoonotic sporotrichosis has spread throughout the Brazilian territory [11,12]. In addition, other countries are also reporting cases of zoonotic sporotrichosis transmitted by naturally infected cats [13,14,15]. The immune system of cats is not able to adequately combat *Sporothrix*, contributing substantially to the increase in the number of sporotrichosis cases among the animals [16], and consequently, increasing cat-to-human transmission, which mostly occurs in housewives [11].

Independently of the transmission form, sporotrichosis is caused by a thermodimorphic species of the *Sporothrix* genus, which under laboratorial culture at 25–30 °C or in environmental niches, grows in the filamentous form, whereas it grows in a yeast-like form in parasitism or when cultivated on enriched culture media at 35–37 °C [1,5,7]. At least 50 species have been described in the *Sporothrix* genus, but the vast majority are saprobiotic [17]. Some species have been described as pathogenic to humans and other mammals, mainly *Sporothrix brasiliensis*, *Sporothrix schenckii* and *Sporothrix globosa* [18,19]. 

*Sporothrix* spp. has been isolated from environmental samples in several countries worldwide [13,20,21,22]. Recently, a systematic review described ecological determinants of the sporotrichosis agents, mainly *S. schenckii*, such as growth in soils with temperatures ranging from 6.6 °C to 28.84 °C and humidity ranging from 37.5% to 99.06%. Moreover, *Sporothrix* spp. is highly associated with decomposition of organic material, which potentially increases its ability to proliferate [20]. In Brazil, a study aiming to isolate and/or identify sporotrichosis agents in soil samples from a geographic area with reports of sporotrichosis cases was unsuccessful [23]. 

To improve our knowledge about the relationship of *Sporothrix* spp. with the environment and the suitability of different molecular methods of *Sporothrix* DNA detection, we applied classical and molecular methods to detect *Sporothrix* spp. in soil samples collected in two cities within the sporotrichosis hyperendemic area in the Rio de Janeiro state, Brazil, where *Sporothrix* spp. has not yet been associated with environmental samples.

## 2. Materials and Methods

### 2.1. Sample Collection and Processing

Soil samples were obtained in Seropédica (22°46′52.3′′ S, 043°45′27.1′′ W) and Nova Iguaçu (22°40′37.3′′ S, 043°24′17.7′′ W), both cities located in Rio de Janeiro state, Brazil. The samples were taken in rural areas where *Paracoccidioides brasiliensis* was recently described [24]. Approximately 100 g of soil were collected using sterile shovels, and stored in sealed sample collection bags (Nasco Sampling/Whirl-Pak^®^, Madson, WI, USA). Samples were processed for culture within 24 h after the collection. One gram of each soil sample was diluted in 9 mL sterile saline, vortexed for 10 min and particles were allowed to settle for 5 min. Serial ten-fold dilutions of the upper homogenous suspensions were plated on Potato Dextrose Agar (PDA) Mycosel Agar (Becton, Dickinson and Company, Sparks, MD, USA) and incubated at 25 °C for 60 days. Putative *Sporothrix* colonies were submitted to micro- and macromorphological tests to confirm genus identification [1].

### 2.2. Soil DNA Extraction

DNA extraction was performed as described [24] using the DNeasy^®^ PowerSoil^®^ Kit (Qiagen, Hilden, Germany), following the manufacturer’s instructions.

### 2.3. Nested-PCR

Nested-PCR was performed in order to detect low amounts of *Sporothrix* DNA [25,26]. The reaction mixture consisted of a total volume of 25 µL, with final concentrations of 10 mM Tris-HCl (pH 9.0), 50 mM KCl, 1.5 mM MgCl_2_, 0.4 µM concentrations of primers (Table S1), 1.5 U of *Taq* DNA polymerase (Invitrogen, Waltham, MA, USA), and 200 µM concentration of each dNTP (Invitrogen). For the first reaction, 4 µL of total extracted soil DNA was used and for the second, 4 µL of the first amplicon. Reactions were as follows: 95 °C for 5 min, 35 cycles of 95 °C for 30 s, 60 °C for 45 s, 72 °C for 30 s and 72 °C for 10 min [26]. The positive control was performed including the DNA of a reference strain for *S. brasiliensis* (CBS 120339), and ultrapure water (Gibco, Walthan, MA, USA) as negative control. As a positive result, an amplified fragment with 152 bp was expected, compared to a 1 kb plus DNA ladder (Invitrogen).

### 2.4. Species-Specific PCR

Species-specific PCR was performed on the environmental DNA samples for the three main relevant species. The reactions (final volume of 25 µL) were obtained using 3 mM MgCl_2_, 400 mM each deoxynucleotide triphosphate, and 50 U/mL *Taq* Polymerase (Invitrogen); 9.5 μL water, 1 μL (10 pmol/μL) of forward and reverse primers (Invitrogen) described in Table S1 and 4 μL of target DNA (25 ng/μL). The reactions were performed as described [27].

### 2.5. DNA Sequencing and Phylogenetic Analyses

The calmodulin amplicons obtained from the species-specific PCR products as well as the nested-PCR products were excised from the gel, purified with the IllustraTM GFXTM PCR DNA and Gel Band Purification Kit (GE Healthcare, Buckinghamshire, UK) and sequenced. Amplified fragments were purified with the QIAquick PCR Purification Kit (QIAGEN, Hilden, Germany), and sequenced at the Fiocruz Technological Platforms Network, Rio de Janeiro, Brazil [28]. The sequences were edited with the Sequencher Software Package version 4.9 (GeneCodes Corporation, Ann Arbor, MI, USA) and aligned with MEGA software version 10 [29]. The sequences were used for the phylogenetic analysis with sequences deposited in the GenBank database. A maximum likelihood (ML) tree was created using 1000 bootstrap replicates. The same dataset was used within DNAsp 5.10 (Universitat de Barcelona, Barcelona, Spain) [30] to calculate the distribution and diversity of haplotypes and the median-joining network was built and visualized using Network Software (Version 5.0.1.0, Fluxus Technology Ltd., Stanway, UK) [31].

## 3. Results

Eighteen soil samples were included in this study, nine from Seropédica and nine from Nova Iguaçú. After 60 days of incubation at 25 °C, fungal growth compatible with *Sporothrix* spp. did not occur in any of the soil samples from either city.

**Figure 1:**
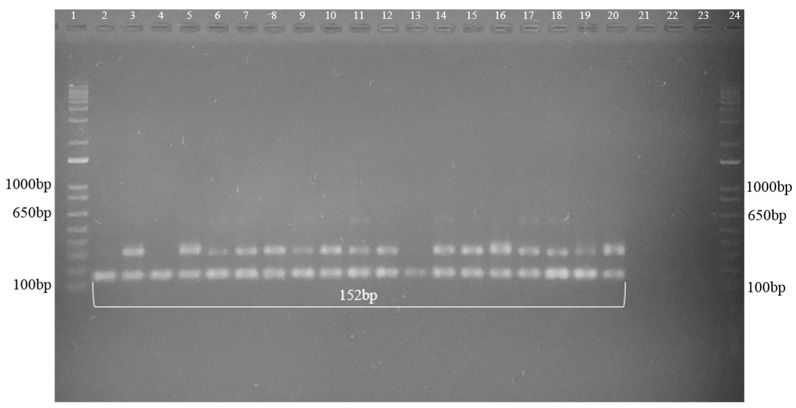
Representative nested-PCR agarose gel of the DNA extracted from soil samples, demonstrating 16 amplified fragments with the amplification of 152 bp fragments. 1 and 24 = Molecular Weight (1 kb Plus–Invitrogen), 2 to 10 = samples from Seropédica, and 11 to 19 = samples from Nova Iguaçu, 20 = Positive control *S. brasiliensis* (IPEC 16490) and 21, 22 and 23 = Negative control.

The nested-PCR demonstrated the presence of DNA amplicons of 152 bp in all 18 studied samples (Figure 1).

The BLAST analysis of the amplified DNA obtained in the nested-PCR (GenBank sequences ON306930 to ON306947) demonstrated a nonspecific alignment with *Sporothrix* spp. Individual sequences showed a high similarity (100%) and query cover (100%) with several fungi from the Ophiostomataceae family, such as *Grosmannia crassivaginata*, *Raffaelea canadensis*, *Ophiostoma nigrocarpum*, *Ophiostoma ambrosium*, *Leptographium huntii* and *Grosmannia serpens*, most of them phytopathogenic and geophilic (Figure S1).

**Figure 2:**
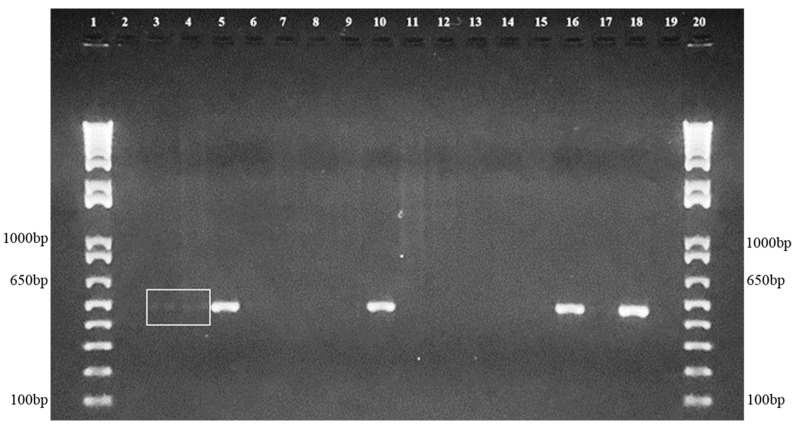
Representative agarose gel of the positive soil samples, demonstrating species-specific amplification of 469 bp fragments compatible with *S. brasiliensis*. 1 and 20 = Molecular Weight (1 kb Plus–Invitrogen), 2 to 10 samples from Seropédica and 11 to 17 samples from Nova Iguaçu, 18 = Positive control *S. brasiliensis* (IPEC 16490) and 19 = Negative control. The white square highlights two positive samples with low-intensity bands.

The PCR using species-specific primers demonstrated the absence of *S. schenckii* and *S. globosa* DNA. However, amplification with *S. brasiliensis* species-specific primers identified this pathogen’s DNA in soil samples from Seropédica (*n* = 4) and Nova Iguaçu (*n* = 1), as depicted in Figure 2.

**Figure 3:**
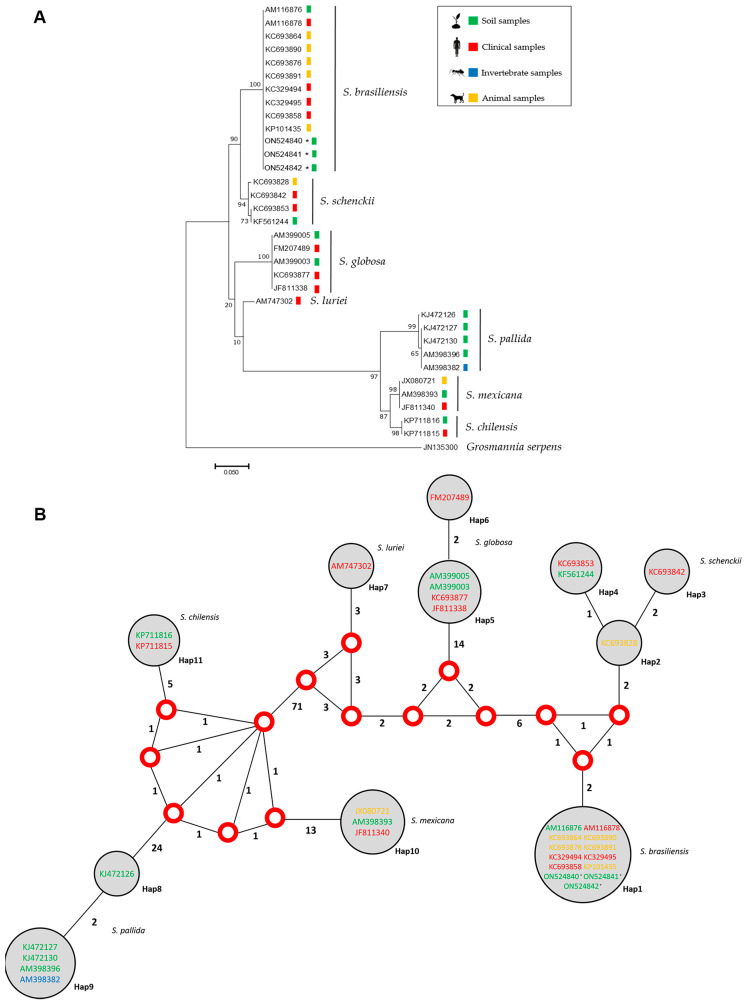
(**A**) Maximum likelihood phylogenetic relationships based on calmodulin partial sequencing between members of clinical, animal and environmental isolates from the *Sporothrix* species with clinical interest. The colored squares represent the origin source of each isolate/sequence. (**B**) Median vector haplotype network demonstrating the phylogenetic relationships among the 33 isolates/sequences of the present study, demonstrating that, in both cases, the three sequences obtained grouped with *S. brasiliensis*. Red dots represent median vectors. Circle size is proportional to the frequency of isolates (h). The numbers around each vertex represent the amount of mutations separating each haplotype and the different colors in the Genbank code represent the isolate/sequence origins. * Indicate sequences generated in this work.

Phylogenetic analysis and haplotype network performed with the sequences obtained from the amplification of the species-specific primer (GenBank sequences ON524840 to ON524842, respectively, for samples 5, 10 and 16 in Figure 2) and sequences deposited in GenBank demonstrated that the three sequences from this study grouped with other sequences of *S. brasiliensis*, including isolates from clinical, animal and environmental sources (Figure 3).

**Figure 4:**
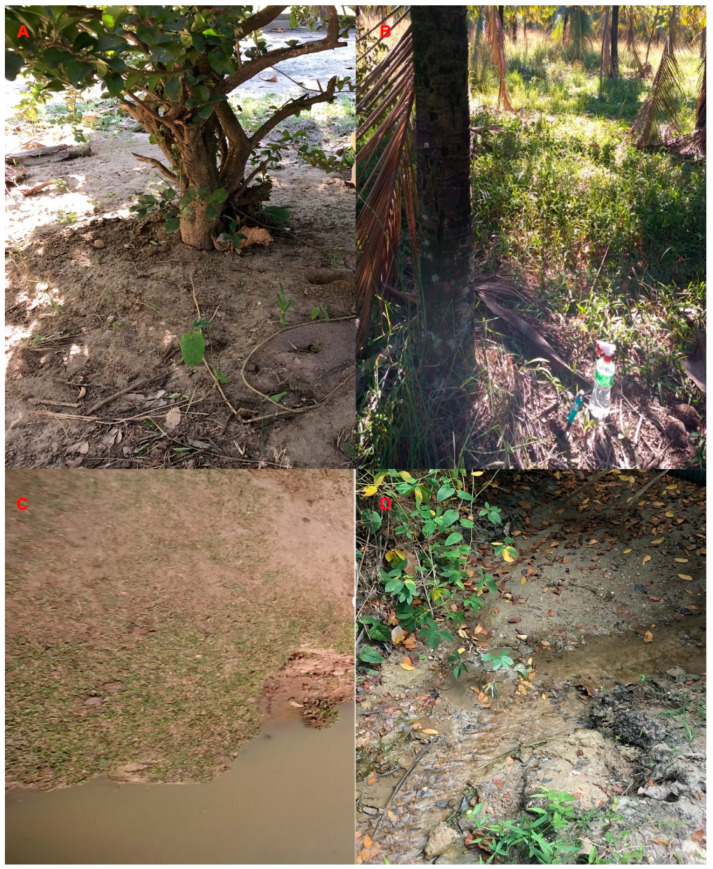
Soil samples with *Sporothrix brasiliensis* DNA detection. (**A**) Soil around an acerola fruit tree (*Malpighia emarginata*). (**B**) Soil around a coconut tree (*Cocos nucifera*) enriched with cow dung. (**C**) Soil around an artificial lake. (**D**) Soil from the bottom of a stream. Sites A to C are located in Seropédica. Site D is located in Nova Iguaçú.

Figure 4 shows the sites where the positive samples for *S. brasiliensis* DNA were collected. They comprise the soil around an acerola fruit tree (*Malpighia emarginata*), around a coconut tree (*Cocos nucifera*) and enriched with cow dung, around an artificial lake, and from the bottom of a stream. An additional positive sample was collected from a soil sample with a spider web.

## 4. Discussion

Over the past two decades, despite a significant increase in research on sporotrichosis and its agents [32], there have been few studies focusing on the isolation and/or molecular identification of *Sporothrix* spp. from environmental sources. Initially, sporotrichosis was known as gardener’s disease due to its association with horticulture-associated injuries [1]. The zoonotic epidemic in Brazil, primarily driven by *S. brasiliensis*, has led to a major shift in the mechanisms for disease acquisition, as traumatic inoculation is now associated with scratches and/or bites from cats [11]. However, the interaction of cats—and other animals—with soil and the transmission of *Sporothrix* to and/or from the environment has been poorly explored, which has resulted in an important knowledge gap in the pathogenesis of cat-associated sporotrichosis. 

Soil is the ecological niche of several human pathogens, including thermodimorphic fungi, such as *P. brasiliensis*, *Coccidioides posadasii* and *Histoplasma capsulatum* [33,34]. The relationship of these fungi with environmental sources is better described when compared to *Sporothrix* spp. [35]. For example, *H. capsulatum* is primarily present in soils with high concentrations of nitrogen compounds, especially those with bird excreta and bat guano [36]. Here, we present some soil characteristics that may improve the knowledge about *S. brasiliensis* environmental habitat. The soil samples where *S. brasiliensis* DNA was detected did not receive direct solar light and/or present high humidity. This last characteristic was previously described for other *Sporothrix* species [20]. Moreover, urban sporotrichosis due to *S. brasiliensis* usually occurs in areas with low infrastructure and precarious sanitation, usually near polluted water sources [37,38].

Some factors may influence the isolation of pathogens from the environment. There are many other fungal species that have faster growth rates in culture that limit the growth of the fungus of interest, which could influence the lack of success in the isolation of *Sporothrix* spp. Recently, our group reported the isolation of a *S. brasiliensis* fungal colony from a wood sample present in a house were several cats with sporotrichosis lived [39]. The presence of sick cats, with high fungal burden in their lesions around this material probably contributed to increase the number of fungal cells present in the wood, which may have facilitated the culture isolation. Both cities from which we collected soil samples report zoonotic sporotrichosis cases [37]. As we were looking for the habitat of *S. brasiliensis*, we chose to collect the materials from an area with some rural characteristics, such as the presence of trees, small rivers and moist soil, among others. We did not observe cats in the area where samples were collected during the harvest of soil samples. In the present study, we consider the possibility of contamination of the environmental material with *S. brasiliensis* by infected cats to be very low.

To overcome the difficulties with isolating fastidious thermodimorphic fungi from the environment, molecular methods are increasingly being used [24,33,40]. An important factor of success is the method used for fungal detection, and in other models, such as *P. brasiliensis*, the most effective technique is nested-PCR [24,41]. Nested-PCR drastically increases the possibility of a successful detection as the number of DNA amplification cycles is high, making it possible to amplify small quantities of DNA that would not be detected by conventional PCR. Nested-PCR yielded positive results in 18 samples; however, sequencing results demonstrated unspecific amplifications with other Ophiostomataceae for the nested-PCR targeting the ITS region. The members of this family are traditional saprobes that are frequently found in soil samples [42]. Nested-PCR has been successfully used in clinical samples, where Ophiostomataceae members besides *Sporothrix* spp. are unlikely to occur [25,26]. The present study demonstrates that this methodology is not appropriate for environmental studies, due to the frequent amplification of DNA from saprobe fungi. 

Real time-PCR has been reported as superior to nested-PCR to detect *H. capsulatum* in environmental samples [43]. The species-specific PCR used in our study was developed for clinical diagnosis purposes [27]. When we applied this method to environmental samples, we identified five positive samples and two patterns of amplifications were observed. The soil samples with high DNA amplification harbored *S. brasiliensis* by sequencing and subsequent phylogenetic analyses. The two samples that presented low amplification could not be sequenced, due to the small quantity of DNA. Nevertheless, there is a high possibility that these faint bands represent small amounts of *S. brasiliensis* DNA. However, as we observed with the nested-PCR, we cannot rule out the possibility of non-specific amplification of other close related fungi, since only members of the genus *Ophiostoma*, among all Ophiostomataceae family, were tested for cross-reaction in the standardization of the method for *Sporothrix* DNA amplification using species-specific primers.

The paucity of studies associating *S. brasiliensis* with the classic environmental transmission of sporotrichosis tends to suggest that this species is exclusively transmitted through the zoonotic pathway. However, *S. brasiliensis* has been isolated from patients with sporotrichosis who did not have contact with infected cats [44,45]. Moreover, *S. brasiliensis* has been isolated from soil in Argentina [46]. In Brazil, the largest environmental study attempting to detect *S. brasiliensis* was unsuccessful in both the isolation and detection of its DNA in 101 samples studied [23]. However, the isolation of *S. schenckii* was demonstrated in soil samples from armadillo burrows. Although *S. schenckii* was not isolated after direct plating of the soil in culture media, it was grown form organs of mice and hamsters inoculated with sand from the armadillo burrow [47]. An environmental relationship of *S. brasiliensis* was observed through fungal isolation from a sample of cat feces buried in sand in São Paulo state, Brazil [48].

Undoubtedly, cats play a major role in zoonotic sporotrichosis transmission. In contrast with the soil, which appears to have a low number of fungal cells, these animals have increased numbers of *S. brasiliensis* parasitic cells in their lesions and they may carry the fungus on their claws, which can traumatically inoculate the fungus into humans and other cats [49]. However, it is noteworthy that the close relationship of these animals with natural sources likely contributed to the emergence and persistence of *S. brasiliensis* as a feline and human pathogen. The strong epidemiological relationship reported between cats and sporotrichosis has incorrectly led to the belief that *S. brasiliensis*-caused sporotrichosis is only zoonotically transmitted, ignoring the original association of sporotrichosis with environmental sources [8]. From the authors’ point of view, it is principally the human–cat interaction that increases the odds of traumatic inoculation of the fungus, as these animals frequently seek human contact and harbor a high fungal burden. On the other hand, the lower amounts of *S. brasiliensis* in soil compared to naturally infected cats makes the classic transmission of *S. brasiliensis* via traumatic injury with vegetation or soil more difficult, but not inconceivable. Furthermore, the sporotrichosis expansion in Rio de Janeiro is caused by a clonal species [50] and, in this study, we did not observe phylogenetic differences among clinical or environmental *S. brasiliensis* strains.

## 5. Conclusions

This study demonstrates the environmental detection of *S. brasiliensis* DNA in a hyperendemic area of sporotrichosis, primarily due to *S. brasiliensis*. This reinforces the initial historical association of sporotrichosis as a disease related to soil and organic material. The detection of *Sporothrix* spp. in environmental samples can be facilitated by implementing existing molecular methods with high sensitivity and specificity. Importantly, sequencing of PCR amplicons must be used to confirm *Sporothrix* spp. identification. A deeper understanding of the ecological niches of *Sporothrix* spp. is a significant step forward in the efforts to resolve this important public health problem in endemic regions.

## Data Availability

Publicly available datasets were analyzed in this study. These data can be found here: https://www.ncbi.nlm.nih.gov/genbank/ (accessed on 28 April 2022) [51].

## Supplementary Materials

The following supporting information can be downloaded at: https://www.mdpi.com/article/10.3390/jof8060604/s1, Figure S1: BLAST Maximum likelihood phylogenetic relationships of amplified sequences obtained in the nested-PCR, presenting similarity with sequences from 16 different fungal species deposited in the GanBank. Table S1: PCR primers used in this study.
