# Supplementary material for: Beyond Domestic Cats: Environmental Detection of Sporothrix brasiliensis DNA in a Hyperendemic Area of Sporotrichosis in Rio de Janeiro State, Brazil
Source: J Fungi (Basel). 2022 Jun 4;8(6):604. doi: 10.3390/jof8060604 (PMC9224889; doi:10.3390/jof8060604)
Supplement: Supplementary file 1 [file jof-08-00604-s001.zip › Figure S1.pdf]

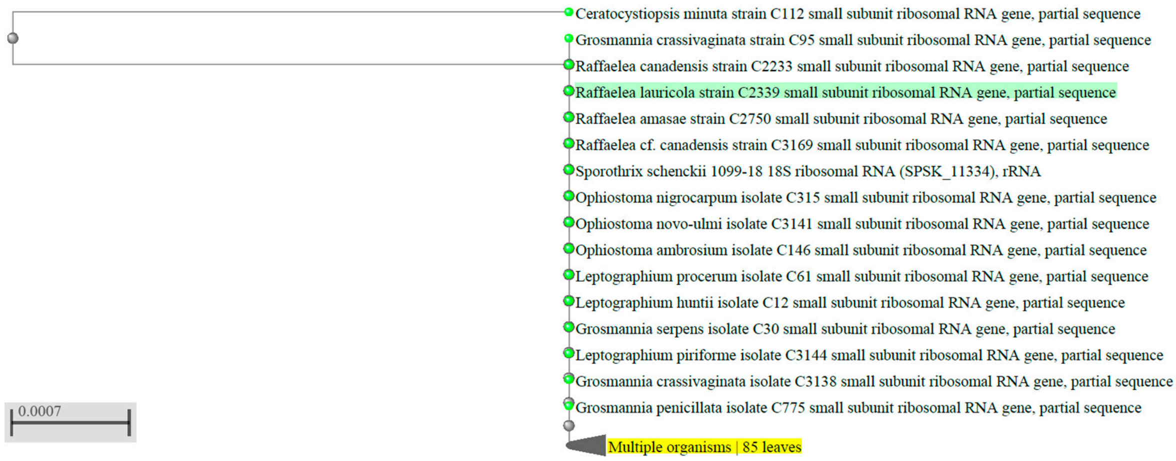

Figure S1: BLAST Maximum likelihood phylogenetic relationships of amplified sequences obtained in the nested-PCR, presenting similarity with sequences from 16 different fungal species deposited in the GanBank
