# Supplementary material for: Beyond Domestic Cats: Environmental Detection of Sporothrix brasiliensis DNA in a Hyperendemic Area of Sporotrichosis in Rio de Janeiro State, Brazil
Source: J Fungi (Basel). 2022 Jun 4;8(6):604. doi: 10.3390/jof8060604 (PMC9224889; doi:10.3390/jof8060604)
Supplement: Supplementary file 1 [file jof-08-00604-s001.zip › Table S1.pdf]

Table S1: PCR primers used in this study

PCR	Primers	Sequence 5' – 3'	Target
Nested - PCR	SS1	CTC GTT CGG CAC CTT ACA CG	*Sporothrix* spp.
	SS2	CGC TGC CAA AGC AAC GCG GG	
	SS3	ACT CAC CAG GTC CAG ACA CGA TG	
	SS4	CGC GGG CTA TTT AGC AGG TTA AG	
Species-specific	Sbra-F	CCC CCG TTT GAC GCT TGG	*S. brasiliensis*
	Sbra-R	CCC GGA TAA CCG TGT GTC ATA AT	
	Ssch-F	TTT CGA ATG CGT TCG GCT GG	*S. schenckii*
	Ssch-R	CTC CAG ATC ACC GTG TCA	
	Sglo-F	CGC CTA GGC CAG ATC ACC ACT AAG	*S. globosa*
	Sglo-R	CCA ATG TCT ACC CGT GCT	
